# Examining the impact of climate change on cereal production in India: Empirical evidence from ARDL modelling approach

**DOI:** 10.1016/j.heliyon.2024.e36403

**Published:** 2024-08-27

**Authors:** Arshdeep Singh, Kashish Arora, Suresh Chandra Babu

**Affiliations:** a Department of Economics and Sociology, Punjab Agricultural University, Ludhiana, Punjab, India; b Indian Council of Agricultural Research – National Institute of Agricultural Economics and Policy Research (ICAR-NIAP), New Delhi, India; c International Food Policy Research Institute, Washington, DC, USA

**Keywords:** Climate change, Cereals, ARDL, FMOLS, Long-run and short-run estimates

## Abstract

Agriculture sector is major sufferer of climate change both at a global level as well as at India level. Cereals account for about 92 % of India's total food grain output and climate change has a significant influence on the production of cereals. This study aimed to evaluate the long-term and short-term effects of climatic and non-climatic variables, specifically temperature, precipitation, cereal area, total cropped area, fertilizer consumption, and pesticide consumption, on cereal production in India. The study included annual time series data that covered the period from 1960 to 2018, covering a period of 58 years. Various econometric techniques were employed to examine these relationships. The validity of a long-term and short-term relationship among the relevant variables included in the study was validated by employing the Autoregressive Distributed Lag (ARDL) technique and the Johansen cointegration test. The ARDL model's estimation outcomes reveals that input factors such as cereal area became a key factor in rising cereal production, as evidenced by its positive coefficient. Similarly, fertilizer consumption and precipitation had positive effects on production in the long run whereas total cropped area and minimum temperature has little influence over the results of production both in short run as well as long run. Furthermore, the long-term findings were also supported using econometric tools like Canonical Cointegrating Regression (CCR) and Fully Modified Least Squares (FMOLS). These methods confirmed that variations in cereal production in India were significantly influenced by both climatic factors and agricultural inputs and factors. The study emphasizes the urgency for policymakers to prioritize proactive measures aimed at reducing the adverse impacts of climate change on cereal production in India. This necessitates a comprehensive strategy integrating sustainable practices, technological innovations, and robust policy frameworks to ensure resilient agricultural sectors and sustainable food production.

## Highlights

• Cereal area, fertilizer and precipitation boosted long-term cereal production.
• Total cropped area and minimum temperature had minimal effect on production over time.
• CCR and FMOLS supported these long-term findings.
• Address climate impacts, ensure sustainable food and boost India's agricultural resilience.

## Abbreviations:

“ARDL -: Autoregressive Distributed Lag
CCR -: Canonical Cointegrating Regression
FMOLS -: Fully Modified Least Squares
LNCP –: Natural Log of Cereal Production
LNCA –: Natural Log of Cereal Area
LNFC -: Natural Log of Fertilizer Consumption
LNPC -: Natural Log of Pesticide Consumption
LNPREC -: Natural Log of Precipitation
LNTMIN -: Natural Log of Temperature Minimum
LNTMEAN -: Natural Log of Temperature Mean
LNTMAX -: Natural Log of Temperature Maximum
ECT –: Error Correction Term
ADF –: Augmented Dickey–Fuller
PP -: Phillips–Perron
VAR -: Vector Autoregression
AIC -: Akaike Information Criterion
SIC -: Schwarz Information Criterion
CUMSUM -: Cumulative Sum
CUMSUMSQ -: Cumulative Sum of Square”

## 1 Introduction

Agricultural sector is main source of income and backbone of developing nations particularly South Asian countries, wherein South Asia with 5 % of the world's agricultural land, feeds 20 % of the world's population [1]. Agriculture's relevance in South Asian countries and regions can be gauged by the substantial proportion of its people, approximately 70 %, residing in countryside where agriculture is the principal source of revenue, the agricultural industry [2,3]. During the 1960s, India had a “green revolution” that allowed it to become independent in terms of food grains and become a net exporter of a variety of agricultural products [4]. The agricultural sector in India has played a crucial role in reduction of poverty and creation of work opportunities [[5], [6], [7]] and provided employment to 55 % of the workforce and 67 % of the population in farming and allied industries [[8], [9], [10]]. The enhancement of agricultural production has been achieved by several strategies, such as advancements in technology, heightened use of fertilizers, the adoption of improved seeds, and the expansion of cultivation areas [[11], [12], [13]]. The drivers in concern have demonstrated enhanced productivity; nevertheless, they have also been associated with substantial alterations in climate, which in turn have a notable impact on both the environment and agricultural output [14]. Therefore, it is of significant interest to investigate the effects of both climatic and non-climatic elements on developing nations such as India.

Climate change is now a significant concern in both developing and developed nations [[15], [16], [17], [18]] and agriculture is anticipated to be the sector most affected by it since weather is a crucial component of agricultural productivity [[19], [20], [21]]. Global land area and agricultural productivity are impacted by climate change [[22], [23], [24]] and inevitably alter the price levels [25,26] in several ways that worsen the nation's food security, including variations in the country's yearly rainfall, average temperature, frequency of heat waves, etc. [27,28].

Many people worldwide consume cereal grains like wheat, maize, and rice; hence they are regarded as fundamental crops [23]. The food security and way of life of rural communities are under risk due to the extreme vulnerability of all these crops to climate change [29,30]. Evidence reveals that the Indian agricultural industry is sensitive to climate change and natural disasters since there is not enough arable land, a sizable population depends on agriculture, rain is necessary for agricultural operations, and there is insufficient technology for adjusting to climate change [31,32]. As cereals accounted for about 92 % of India's total food grain output in the years 2022–2023, climate change has a substantial influence on the production of cereals. Additionally, the production of cereals makes up almost one-third of the total calories consumed in South Asian nations [33] making it a crucial component of the food security of these economies [34]. According to the most recent climate change predictions, temperatures will rise by 2–4 °C, there will be an increase in rainfall throughout the rainy season, and precipitation will climb by 15–20 % physically [35]. It will also have an influence on agricultural output, as shown by the fact that production of cereal, rice, cotton, sugarcane, sunflowers, and wheat drastically dropped [35,36].

Literature extensively examines the effect of climate change on individual cereal crops (such as rice, wheat, maize, etc.), but few limited studies focus on aggregate cereal production (sum of all cereal crop production) in India. The present study investigates the combined effect of climatic factors (rainfall and temperature) and non-climatic factors (cropped area, fertilizer consumption, and pesticide use) on aggregate cereal production in India. It aims to address the lack of comprehensive empirical evidence on these influences.

## 2 Literature review

Several recent studies have delved into the complex relationship between climatic conditions and cereal production across different regions, shedding light on both global and localized implications for food security. The rainfall impacts cereal crops positively, while average temperatures typically pose challenges, particularly in Africa [37]. Significant differences were found between country-specific scenarios regarding the impact of climate conditions on cereal output [37]. Climatic factors, such as carbon dioxide (CO_2_) levels and average temperatures, are found to have a detrimental effect on both agricultural value-added and cereal production. Furthermore, the influence of financial development on the output of cereals and agricultural value-added curves an inverted U shape. On the other hand, factors like cropped area, income level, and the size of the rural labor force are observed to positively contribute to production of cereals and agricultural products with added value [38]. In East Africa, the rainfall and carbon emissions showed favorable long-run effects, average temperatures had negative repercussions, particularly in the short run. In addition, elements like the amount of cultivated land and the population in rural areas contributed positively to the increase in agricultural output [39]. Moreover, the detrimental effects of several factors on wheat production, apart from land used for cereal cultivation, which had both short- and long-term benefits [40]. Another study from Pakistan reported that the rising temperatures had a significant negative long-term effect on the production of major crops such as wheat, rice, and cotton in Pakistan from 2000 to 2019, with an insignificant short-term impact [41]. Additionally, the area under cultivation and fertilizer input positively affected crop production in both the long and short run [41,42]. The study examined the effects of climate change elements and inventive capacities on food production in Algeria. The findings indicated that while temperatures had a negative correlation with food production, precipitation, human capital, and physical capital all had favorable effects [43]. The implications of climate change on India's food production, highlighting that energy use, rainfall, and methane emissions enhance food productivity, while temperature and N_2_O emissions hinder it in both the long and short run, with CO_2_ emissions having no effect [44]. Economic and environmental factors are crucial for Turkey's cereal supply, with energy use positively impacted by cereal output, climate change has varying effects and CO_2_ emission having a negatively impact [45]. Average temperature has a negative impact on Somalia's sorghum production, while rainfall and the amount of land used for sorghum farming have shown positive impact on yield [46]. In Bangladesh, precipitation has positive influence while carbon di-oxide and temperature have adverse impact on cereal production [1]. Furthermore, in India the rainfall has positive impact on rice crop productivity and small increase in temperature leads to decline in agricultural productivity [47]. Some crops like jowar, groundnut, sugarcane, gram, sesame, and bajra exhibited a positive impact of temperature production and certain crops, including tea, cotton, rice, wheat, and arhar are sensitive to climate conditions [32].

Despite extensive research on the effects of climate change on individual cereal crops such as wheat, rice, maize, barley, sorghum, millet (including bajra), oats, rye [3,[30], [31], [32],^41^,^42^,[48], [49], [50], [51], [52], [53], [54], [55], [56], [57], [58], [59], [60], [61], [62]] there is a notable gap in studies examining the aggregate cereal production across global [1,37,39,40,45,[63], [64], [65], [66], [67], [68], [69], [70], [71], [72], [73], [74], [75], [76]] and very limited in India [77]. Furthermore, the influence of non-climatic factors such as cropped area, fertilizer consumption, and pesticide use on cereal production remains insufficiently addressed. The combined effects of climatic and non-climatic factors on cereal yields lack comprehensive empirical evidence, and the specific impacts of rainfall, temperature, and agricultural inputs on cereal yields are not well understood.

The present research work closes this gap by analyzing the short- and long-term impacts of climatic variables on cereal production using the ARDL model. This method allows for a more in-depth analysis of the factors effecting cereal production. This study is distinctive from others as we employed nine variables, including input and climate factors over a long-time span.

## 3 Data and methodology

### 3.1 Data

The purpose of this study was to evaluate both the short and the long-term impact of climatic factors and agricultural inputs on cereal production in India. The analysis employed 58-year time-series datasets that span the years 1961–2018. The dependent variable in the study, cereal production (CP), was examined along with independent variables like the area cultivated with cereal crops (CA), fertilizer and pesticide consumption (FC and PC), and climatic variables like average precipitation (PREC), minimum, mean and maximum temperatures (TMIN, TMEAN, and TMAX). The minimum, mean, and maximum temperature data were obtained from Climate Change Knowledge Portal [78] while the remaining variables' data were obtained from the website of Reserve Bank of India [79]. All data pertaining to various variables were gathered on an annual basis. The minimum and maximum temperature data were aggregated to annual figures from the mean of monthly means obtained from daily data. Similarly, rainfall data was transformed into annual values, as outlined in Table 1, and subsequently converted into natural logarithmic values. Table 1 presents a comprehensive summary of the variables incorporated in the study, along with their respective sources, rationale, and descriptions of the data.

**Table 1:** List of study variables selected for the analysis.

**Variable type**		**Variables**	**Abbreviation**	**Units**	**Data source**
Output variable	Dependent Variables	Cereal production	LNCP	Million ha	[1]
Input variables	Independent Variables	Cereal Area	LNCA	Million tonnes	[1]
		Total Cropped Area	LNTCA	thousand ha	[1]
		Fertilizer consumption	LNFC	Lakh tonnes	[1]
		Pesticide consumption	LNPC	thousand tonnes	[1]
Climatic Variables		Precipitation	LNPREC	millimeter	[14]
		Min Temperature	LNTMIN	°Celsius	[14]
		Mean Temperature	LNTMEAN	°Celsius	[14]
		Max Temperature	LNTMAX	°Celsius	[14]

The rationale for using these variables in our study, as outlined in Table 1, was as follows: Several scholars have investigated the relationship between climatic factors and crop production across various global regions [53,58,66,69]. Previous findings indicate that average temperature negatively impacts crop production [66,80], whereas rainfall is anticipated to have a positive influence [50,58]. Our study includes mean temperature [52,65,74], minimum temperature [56,81] and maximum temperature [56,82] due to their direct impact on crop yield [80,83,84]. Moreover, total cropped area [82,85], fertilizer [51,52,60,86,87] and pesticide consumption [52,60,86,88] have been integrated into previous models by various researchers. Many studies on the effects of climate change on crop production have employed the ARDL approach, typically focusing on 4–5 variables over shorter periods (35–40 years), with few examining 9 variables over longer time series (>50 years) [48]. Our study sets itself apart by incorporating nine variables spanning input, climatic, and financial aspects over an extended period [48]. To our knowledge, no prior research has examined these variables collectively within a single study in India.

### 3.2 Methodology

The association between climate change factors and agriculture variables was examined using the following functional model:

(1) CP=(CA,TCA,FC,PC,PREC,TMIN,TMEAN,TMAX)

Equation (1) can be rephrased as follows by taking natural logarithm on both sides of the equation. LNCP, LNCA, LNTCA, LNFC, LNPC, LPREC, LTMIN, LNTMEAN and LTMAX stand for natural logarithm of cereal production, cereal area, total cropped area, fertilizer and pesticide consumption, minimum, mean and maximum temperature, respectively.

(2) $$LNCP=\beta_{0}+\beta_{1}\text{LNCA}+\beta_{2}\text{LNTCA}+\beta_{3}\text{LNFC}+\beta_{4}\text{LNPC}+\beta_{5}\text{LNPREC}+\beta_{6}\text{LNTMIN}+\beta_{7}\text{LNTMEAN}+\beta_{8}\text{LNTMAX}+\varepsilon_{t}$$

#### 3.2.1 Econometric technique

The use of a suitable method for gathering empirical data is essential to evaluate the impact of climate change on India's cereal production. An array of studies from the literature that used various cointegration approaches tailored to different scenarios were used in the empirical evaluation. When compared to the aforementioned cointegration strategies, the ARDL method excels in many ways. Prior studies have favored the cointegration technique developed by Johansen and Juselius [89] for testing the cointegration relationships between the selected variables, and the current study adopted a similar strategy. As a result, it is crucial to arrange the variables in a consistent order to ensure that this methodology is applied correctly.

This study uses the ARDL method, also known as the “Bound Test,” which was developed by Ref. [90] after being first proposed by Pesaran and Shin [91]. The ARDL methodology offers several significant advantages over conventional cointegration methods like Engle and Granger [92] or Johansen [93,94]. As opposed to earlier methodologies that demanded extensive time series data, it is particularly well-suited for the analysis of datasets with small sample sizes [95]. In addition, the ARDL approach exhibits remarkable flexibility in terms of the order of variable integration [51] and has the ability to estimate long-run and short-run cointegration regardless of whether the variables are integrated at order I (0), I (1) or the combination of both [90,96,97]. The ARDL approach also permits the simultaneous regression of long-run and short-run cointegration [58,98]. Notably, this analysis accounts for potential variations in the adjustment process by taking the possibility of asymmetry in the conditional error correction coefficients into account [46,58]. A bias-corrected bootstrap method is used to ensure accurate statistical inferences regarding the long-run cointegration among the variables of interest, improving the accuracy and reliability of the results [46,58].

#### 3.2.2 Specification of the model

The ARDL cointegrating equations are as follows:

(3) $$\Delta LNCP_{t}=\delta_{0}+\delta_{1}\sum_{i=1}^{p}\;\Delta LNCP_{t-1}+\delta_{2}\sum_{i=1}^{p}\;\Delta LNCA_{t-1}+\delta_{3}\sum_{i=1}^{p}\;\Delta L{NTCA}_{t-1}+\delta_{4}\sum_{i=1}^{p}\;\Delta L{NFC}_{t-1}+\delta_{5}\sum_{i=1}^{p}\;\Delta L{NPC}_{t-1}+\delta_{6}\sum_{i=1}^{p}\;\Delta L{NPREC}_{t-1}+\delta_{7}\sum_{i=1}^{p}\;\Delta L{NTMIN}_{t-1}+\delta_{8}\sum_{i=1}^{p}\;\Delta \mathrm{LN}\;{TMEAN}_{t-1}+\delta_{9}\sum_{i=1}^{p}\;\Delta L{NTMAX}_{t-1}+\lambda_{1}LNCP_{t-i}+\lambda_{2}{LNCA}_{t-i}+\lambda_{3}L{NTCA}_{t-i}+\lambda_{4}L{NFC}_{t-i}+\lambda_{5}\;\mathrm{LN}\;{PC}_{t-i}+\lambda_{6}\;\mathrm{LN}\;{PREC}_{t-i}+\lambda_{7}\;\mathrm{LN}\;{TMIN}_{t-i}+\lambda_{8}L{NTMEAN}_{t-i}+\lambda_{9}\;\mathrm{LN}\;{TMAX}_{t-i}+\varepsilon_{t}$$

Equation (3) represent the short-run dynamics and long-run relationship of the model, where δ_0_ is the intercept, δ_1_ to δ_9_ are the short-run parameters, λ_1_ to λ_9_ are the long-run parameters, and ε_t_ represents the error term.

(4) $$LNCP_{t}=\delta_{0}+\delta_{1}\sum_{i=1}^{p}\;\Delta LNCP_{t-1}+\delta_{2}\sum_{i=1}^{p}\;\Delta \mathrm{LN}\;CA_{t-1}+\delta_{3}\sum_{i=1}^{p}\;\Delta \mathrm{LN}\;{TCA}_{t-1}+\delta_{4}\sum_{i=1}^{p}\;\Delta L{NFC}_{t-1}+\delta_{5}\sum_{i=1}^{p}\;\Delta L{NPC}_{t-1}+\delta_{6}\sum_{i=1}^{p}\;\Delta L{NPREC}_{t-1}+\delta_{7}\sum_{i=1}^{p}\;\Delta L{NTMIN}_{t-1}+\delta_{8}\sum_{i=1}^{p}\;\Delta L{NTMAX}_{t-1}+\delta_{9}\sum_{i=1}^{p}\;\Delta L{NTMAX}_{t-1}+\varphi {ECT}_{t-i}+\varepsilon_{t}$$

The residuals that were produced by Equation (4) are ECT_t-1_ and reflects the rate of adjustment in the direction of the long-term equilibrium. After establishing long-run cointegration, we analyzed the long-run coefficients using FMOLS and CCR techniques to ensure robustness. Diagnostic tests validated the ARDL model: the Jarque-Bera test confirmed residual normality; Breusch-Godfrey test indicated no autocorrelation; Breusch-Pagan-Godfrey, ARCH, Glesier and Harvey test established heteroskedasticity. However, Ramsey Reset test confirmed correct specification, and CUSUM and CUSUM squares tests affirmed parameter stability.

## 4 Results and discussion

### 4.1 Descriptive analysis

The goal of this study was to investigate the effects of agriculture inputs and climate change on India's annual cereal production. Table 2 presents the results of the descriptive analysis of the variables used in the model. The mean values range from 4.959 (LNCP) to 12.097 (LNTCA), with medians largely aligned. The maximum values vary between 5.573 (LNCP) and 12.211 (LNTCA), while the minimum values range from 1.509 (LNFC) to 4.134 (LNCP). Standard deviations are generally low, with values between 0.021 (LNTMIN) and 1.133 (LNFC). Skewness indicates slight deviations from normality, with the most skewed being LNFC at −1.497. Kurtosis values suggest mild tail behavior, ranging from 1.871 (LNTCA) to 4.544 (LNFC). Jarque-Bera tests highlight departures from normality, where LNFC exhibits the highest value at 27.417. Notably, LNPREC has a skewness close to zero (0.057) and the lowest Jarque-Bera value (0.859), indicating relatively normal distribution. Probability values for the Jarque-Bera tests reinforce these observations. The data comprises of 58 observations for each variable.

**Table 2:** Descriptive statistics of the variables.

**Particulars**	**LNCP**	**LNCA**	**LNTCA**	**LNFC**	**LNPC**	**LNPREC**	**LNTMIN**	**LNTMEAN**	**LNTMAX**
Mean	4.959	4.604	12.097	4.401	3.744	7.006	2.922	3.202	3.421
Median	5.058	4.610	12.113	4.809	3.886	7.014	2.922	3.202	3.421
Maximum	5.573	4.679	12.211	5.683	4.329	7.160	2.969	3.238	3.451
Minimum	4.134	4.526	11.953	1.509	2.245	6.773	2.878	3.170	3.398
Std. Dev.	0.411	0.036	0.077	1.133	0.496	0.087	0.021	0.015	0.011
Skewness	−0.353	−0.567	−0.321	−0.881	−1.497	−0.259	0.057	0.248	0.262
Kurtosis	1.958	2.868	1.871	2.796	4.544	2.703	2.410	2.639	2.811
Jarque-Bera	3.823	3.149	4.081	7.612	27.417	0.859	0.873	0.908	0.752
Probability	0.148	0.207	0.130	0.022	0.000	0.651	0.646	0.635	0.687
Observation	58	58	58	58	58	58	58	58	58

### 4.2 Correlation among variables

The correlation matrix between the chosen variables is shown in Table 3. Notably, with correlation coefficients of 0.986 and 0.965, respectively, LNCP exhibits strong positive correlations with LNTCA and LNFC. In addition, a positive correlation of 0.705 exists between LNCP and LNPC. Low coefficients, such as 0.033 with LNCP, indicate that variables precipitation, as represented by LNPREC, have little correlation with other variables. Like the temperature variables, the correlation between LNCP with LNTMIN, LNTMEAN and LNTMAX varies from 0.586 (LNTMAX) to 0.775 (LNTMIN). These conclusions show the intricate relationships between the variables that were examined and help us comprehend how those relationships interact with one another in the context of the dynamics of cereal production in India.

**Table 3:** Correlation matrix of the selected variables.

**Variables**	**LNCP**	**LNCA**	**LNTCA**	**LNFC**	**LNPC**	**LNPREC**	**LNTMIN**	**LNTMEAN**	**LNTMAX**
LNCP	1.000	0.269	0.986	0.965	0.705	0.033	0.775	0.702	0.586
LNCA	0.269	1.000	0.311	0.363	0.671	0.260	0.042	−0.056	−0.163
LNTCA	0.986	0.311	1.000	0.952	0.708	0.111	0.750	0.668	0.544
LNFC	0.965	0.363	0.952	1.000	0.806	−0.091	0.781	0.710	0.595
LNPC	0.705	0.671	0.708	0.806	1.000	−0.063	0.497	0.404	0.278
LNPREC	0.033	0.260	0.111	−0.091	−0.063	1.000	−0.087	−0.161	−0.231
LNTMIN	0.775	0.042	0.750	0.781	0.497	−0.087	1.000	0.977	0.900
LNTMEAN	0.702	−0.056	0.668	0.710	0.404	−0.161	0.977	1.000	0.972
LNTMAX	0.586	−0.163	0.544	0.595	0.278	−0.231	0.900	0.972	1.000

### 4.3 Unit root tests results

The findings demonstrate that the majority of variables exhibit significant evidence of stationarity at the 1 % level for both the ADF and PP tests (Table 4), indicating that these variables are integrated of order zero (I (0)). There is noteworthy evidence of stationarity in both tests for variables like LNCP, LNCA, LNTCA, LNFC, LNPC, LNPREC, LNTMIN, LNTMEAN, and LNTMAX at a significance level of 1 %. Table 4 indicates that the results of both the ADF and PP tests suggest that all variables in this study exhibit stationarity at the first difference I (1). Hence, these tests show the application of the ARDL model that is suitable for use.

**Table 4:** Results of Augmented Dickey Fuller and Phillips Perron unit root test.

**Particulars**	**ADF**		**PP**	
	**I(0)**	**I(1)**	**I(0)**	**I(1)**
LNCP	−2.81*	−5.97***	−0.84	−18.57***
LNCA	−2.22	−7.20***	−3.29**	−13.85***
LNTCA	−1.27	−8.87***	−1.43	−16.19***
LNFC	−4.04***	−3.96***	−7.12***	−5.14***
LNPC	−2.54	−1.88	−3.94***	−7.78***
LNPREC	−8.69***	−5.73***	−8.71***	−24.13***
LNTMIN	−0.69	−3.09**	−2.79*	−16.77***
LNTMEAN	−0.38	−3.32**	−3.43**	−17.66***
LNTMAX	−0.81	−2.90*	−4.10***	−16.70***

**Note:** (*) Significant at the 10 %; (**) Significant at the 5 % and (***) Significant at the 1 % respectively.

### 4.4 Lag selection criteria

**Fig. 1:**
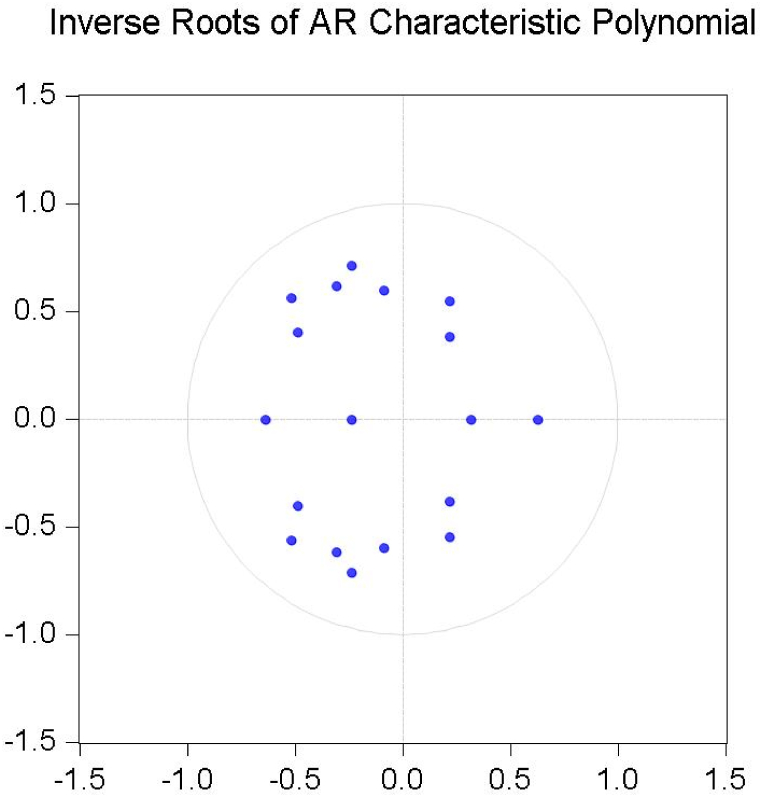
Inverse roots of AR test.

**Fig. 2:**
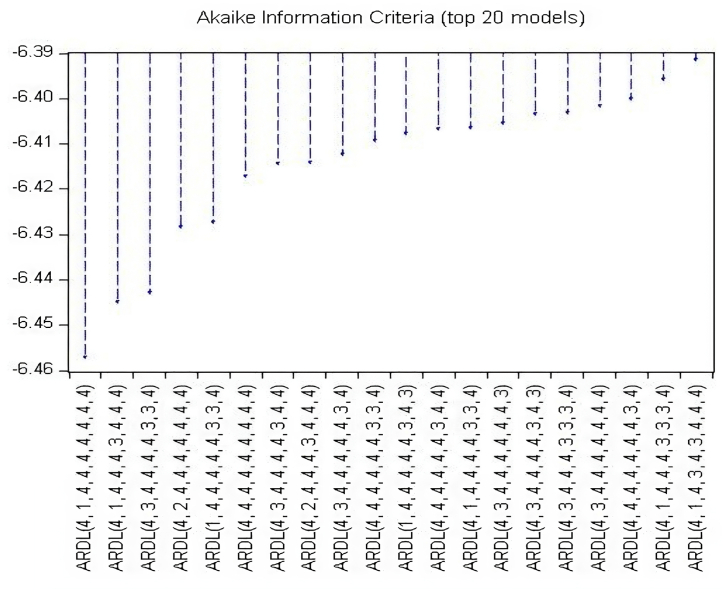
AIC value of top ARDL models.

It is necessary to choose an appropriate lag order for the variables to apply the ARDL bound test to determine out if there is cointegration between cereal production, cereal area, total cropped area, minimum, mean, and maximum temperature, fertilizer and pesticide consumption, and precipitation. For this purpose, the study employed the VAR model's optimal lag order, and the outcomes are shown in Table 5. While there are many lag selection criteria listed in Table 5, this study used popular ones like the AIC and SIC. According to the AIC, lag 4 is the ideal lag value for the cereal production in the model, and Fig. 2 displays the top 20 models chosen based on the AIC. The AIC criterion has also been used in other studies [61,[99], [100], [101]] to establish the number of lag lengths in the ADF test. As shown in Fig. 1, where dots inside the circle symbolize the validation of result accuracy. Additionally, the polynomial graph was utilized to verify the appropriate lag length under the VAR method.

**Table 5:** Lag selection criteria.

**Lag**	**LogL**	**LR**	**FPE**	**AIC**	**SC**	**HQ**
0	1273.291	NA	0.000	−47.709	−47.374^a^	−47.580
1	1365.684	149.922	0.000	−48.139	−44.793	−46.852
2	1439.896	95.214	0.000	−47.883	−41.526	−45.438
3	1541.023	95.403	0.000	−48.642	−39.274	−45.040
4	1732.244	115.454^a^	0.000^a^	−52.802^a^	−40.422	−48.041^a^

**Note: LR:** sequential modified LR test statistic (each test at 5 % level); **FPE:** Final prediction error; **AIC:** Akaike information criterion; **SC:** Schwarz information criterion; **HQ:** Hannan-Quinn information criterion.

a Indicates lag order selected by the criterion.

### 4.5 Results of ARDL bound and Johansen cointegration test

It is crucial to carry out the ARDL bound test [90] to determine the long and short-run relationships between variables to confirm the existence of cointegration. The ARDL bounds method was used in the current study to investigate the long-term relationships between LNCP, LNCA, LNTCA, LNFC, LNPC, LNPREC, LNTMIN, LNTMEAN and LNTMAX. The findings in Table 6 and Fig. 2 show that there is a long-term association. The J-J cointegration technique's results are shown in Table 7, which attest to the variables' effectiveness and robustness over the long term. The LNCP, LNCA, LNTCA, LNFC, LNPC, LNPREC, LNTMIN, LNTMEAN, LNTMAX estimated F-statistics, i.e., 6.46, 5.163, 3.314, 16.191, 4.070, 3.962, 3.873 are exceed the upper threshold of the I (1) value at different significance levels, suggesting the rejection of the null hypothesis (H0)., i.e., “no cointegration among the variables.” While the LNCA, LNPC estimated F-statistics, i.e., 0.997, 2.64 are lower than the lower bound I (0).

**Table 6:** Results of Johansen cointegration test.

**Hypothesized No. of CE(s)**	**Eigen value**	**Trace Statistic**	Prob.**
**Trace**			
None *	0.851	353.417	0.0000
At most 1 *	0.696	250.588	0.0000
At most 2 *	0.651	186.260	0.0000
At most 3 *	0.474	129.485	0.0000
At most 4 *	0.439	94.800	0.0002
At most 5 *	0.392	63.562	0.0009
At most 6 *	0.256	36.650	0.0069
At most 7 *	0.207	20.691	0.0075
At most 8 *	0.140	8.162	0.0043
**Maximum**			
None *	0.851	102.829	0.0000
At most 1 *	0.696	64.327	0.0000
At most 2 *	0.651	56.775	0.0000
At most 3 *	0.474	34.685	0.0000
At most 4 *	0.439	31.238	0.0002
At most 5 *	0.392	26.912	0.0009
At most 6 *	0.256	15.959	0.0069
At most 7 *	0.207	12.529	0.0075
At most 8 *	0.140	8.162	0.0043

Note:***, ** represents the 1 %, 5 % significance level, respectively. **Expose the prob. values of MacKinnon-Haug-Michelis

**Table 7:** The summary of ARDL bounds testing.

**Model for estimation**	**Optimal lag**	**F-stat**
F_LNCP_ (LNCP/LNCA, LNTCA, LNFC, LNPC, LNPREC, LNTMIN, LNTMEAN, LNTMAX)	ARDL (4,1,4,4,4,4,4,4,4)	6.46^a^
Bounds test critical values: K (8)	I (0)	I (1)
1 %	2.62	3.77
5 %	2.11	3.15
10 %	1.85	2.85

a *represents the 1 % significance level. The critical values are based on* [20]. *Here, K represents number of parameters*.

### 4.6 Long-run and short-run estimates

The ARDL long-run and short-run results, as presented in Table 8, reveal significant insights into the connection between climate and agriculture variables and cereal production in India. Expanding cereal crop production is dependent on increasing cereal crop cultivated land area [82]. Further, the increased agricultural carbon emissions, often resulting from excessive chemical use and energy-intensive agro-based technologies, pose a challenge to cereal production growth [76]. Furthermore, multiple researchers have demonstrated that the escalating global warming issue has the potential to impair cereal production worldwide and the global average temperature has risen by approximately 0.5 °C–0.6 °C over the past decades [102]. This temperature shift could result in a 10–15 % decrease in cereal production, resulting in higher market prices [103].

**Table 8:** The ARDL long-run and short-run results.

**Variables**	**Coefficient**	**Std. Error**	**t-stat**	**Prob.**
**Long run**				
LNCA	0.613***	0.075	8.141	0.000
LNTCA	−0.266	0.420	−0.632	0.540
LNFC	0.081***	0.022	3.705	0.004
LNPC	0.019	0.022	0.845	0.416
LNPREC	0.258***	0.082	3.140	0.009
LNTMIN	−0.155	14.499	−0.011	0.992
LNTMEAN	−2.349	38.062	−0.062	0.952
LNTMAX	0.838	23.812	0.035	0.973
C	0.006**	0.003	2.151	0.055
**Short run**				
ΔLNCA	0.843***	0.030	27.895	0.000
ΔLNTCA	0.258*	0.124	2.089	0.061
ΔLNFC	−0.020	0.020	−0.982	0.347
ΔLNPC	−0.048***	0.015	−3.238	0.008
ΔLNPREC	0.172***	0.028	6.164	0.000
ΔLNTMIN	−4.110	3.081	−1.334	0.209
ΔLNTMEAN	8.060	8.243	0.978	0.349
ΔLNTMAX	−4.382	5.225	−0.839	0.420
ECM_t-1_	−1.795***	0.166	−10.837	0.000
R-squared	0.999			
Adjusted R-squared	0.997			
F-statistic	213.114***			
Durbin–Watson stat	1.49			

**Note:** *, **, *** exhibits significance at 10 %, 5 %, 1 % level.

However, the present study identifies a favorable and statistically significant relationship between LNCA and LNCP. The findings show that increasing the LNCA has a direct impact on LNCP in both the short and long term. With a coefficient of 0.613, this means that a 1 % increase in LNCA results in a 0.613 % increase in LNCP over time. These conclusions are consistent with earlier researches by Refs. [54,58,60,62,64,66,69,72,74,75,[104], [105], [106], [107], [108]]. Josephson et al. [109] and Ahsan et al. [69] revealed that with every 1 % increase in area under cereal crops the crop production increase by 0.16 % in long-run. Additionally, the study reveals that a 1 % increase in LNCA correlates with a 0.843 % increase in LNCP in the short term. Moreover, while LNTCA negatively affects LNCP in the long term, it demonstrates a significant positive impact on LNCP in the short term. Specifically, a 1 % increase in LNTCA is projected to decrease LNCP by 0.266 % in the long run while increasing it by 0.258 % in the short run.

Fertilizers are widely thought to boost agricultural productivity by enriching soil nutrients [110]. They improve land fertility and plant growth [50] as a result, fertilizers are expected to have a positive impact on agricultural productivity. This increase in agricultural production has been accomplished through a variety of means, including technological advancements, increased fertilizer utilization, improved seed quality, and expanded cultivation areas. Because cultivable land is limited, future efforts to increase cereal production and productivity are likely to rely on intensive input utilization, particularly fertilizers.

The study findings show that LNFC has a positive and statistically significant influence on LNCP over the long term, with a significance level of 1 %. This means that a 1 % increase in LNFC results in a 0.081 % increase in LNCP in the long run. These findings are consistent with empirical studies conducted by Refs. [60,67,74,87,107,[110], [111], [112], [113], [114], [115]] all of which also found positive correlations between fertilizer application and crop production. Chandio et al. [54] observed that 1 per cent increase in fertilizer consumption could raise agricultural output by 0.38 %. Appropriate usage of fertilizers could improve soil nutrition and soil fertility. The effect of fertilizer consumption on agricultural output is also notable. However, in the short term, this study reveals a contrary result, indicating that estimated LNFC has a negative impact on LNCP. This outcome could be attributed to the inherent fertility of cultivated lands in the short run, where excessive fertilizer application may not only harm crops but also result in runoff into water bodies and groundwater. This runoff has the potential to cause harmful algal blooms that are harmful to ecosystems, humans, and animals, including pets.

LNPC has a positive long-term correlation with LNCP as highlighted in Table 8. This implies that a 1 % increase in LNPC leads to a 0.019 % increase in LNCP over time. Several factors contribute to India's favorable relationship between LNPC and LNCP. Crop and plant diseases are on the rise as the environment deteriorates, forcing farmers to resort to increased pesticide use to protect their crops from insects. This rising demand for pesticides is wreaking havoc on the environment. LNPC has decreased significantly, like LNFC patterns. In this context, a 1 % increase in LNPC results in a 0.048 % decrease in LNCP in the short term. While the short run impact may not be valid, pesticide use in general has intricate ties to environmental degradation, particularly in the long run. Pesticides cumulative effects may contribute to environmental degradation over time. Notably, the multifaceted negative consequences of pesticide application emerge right away. Pesticides seep into the soil in large quantities, compromising soil quality and eventually affecting agricultural productivity. Some of these pesticides make their way into bodies of water via runoff, making the water toxic and harmful to aquatic life. Another substantial portion evaporates into the atmosphere, contributing to global warming. Furthermore, pesticides can harm beneficial insects, including pollinators. The entire process of pesticide transformation and its consequences are inextricably linked to environmental degradation. To mitigate the negative consequences of usage of pesticides and IPM methods emerges as a viable option [88]. IPM is a comprehensive strategy for reducing pesticide toxicity while maintaining agricultural productivity.

Examining other climatic factors, specifically LNPREC, the results show a notable and statistically significant positive impact at the 1 % significance level, both in the long and short run. This means that a 100 mm increase in LNPREC results in an increase in LNCP of about 0.258 % in the long run and 0.172 % in the short run. The enhanced long-term effect compared to the short-term outcome can be attributed to increased average yields, even in the face of consistent LNCA and saturated irrigated regions. LNPREC is a critical determinant in the agricultural sector. This finding is inherently logical, as it emphasizes cereal farming reliance on rainfall/precipitation patterns. The beneficial impact of LNPREC on crop yields aligns with earlier reports by Refs. [53,57,59,60,68,71,75,116,117] which indicated that rainfall enhances agricultural production. The results are in line with the findings of Pickson et al. [66] and He et al. [118], which established that precipitation exerts a substantial favorable influence on China's cereal cultivation. But in case of Somalia, Warsame et al. [58] observed that with 1 % increase in average rainfall and temperature leads to a reduction in crop production by 0.11 % and 8.5 %, respectively in the short run. Minimum, mean, and maximum temperatures all play important roles in cereal production in India. All are required for the plant growth cycle, but only within a certain range; even minor variations can result in crop failure and significant yield losses. The minimum temperature influences germination, early growth stages, and pest dynamics, whereas high maximum temperatures can cause heat stress, impede pollination, and increase water demand. The duration of the growing season, the timing of phenological events, and overall climate resilience are all affected by mean temperature, which is the average of these extremes. Managing these temperature factors collectively is critical for optimal crop growth, yield formation, and adaptation to climate variations, thereby safeguarding the nation's cereal production and the nation's overall food security. The coefficients associated with average LNTMIN have a negative impact on cereal yield in both the long and short run. This means that for every 1 °C increase in LNTMIN, LNCP is expected to fall by 0.155 %–4.110 %. These results underscore the susceptibility of crop productivity to substantial yield declines resulting from climate change and extreme weather occurrences such as floods and droughts in the forthcoming decades [35]. Attiaoui and Boufateh [68] revealed that with every 1 % increase in average temperature the crop production will decrease by 11.5 % in the long run. The IPCC [119] reported in 2013 that even a 1 °C temperature increase could result in a 5 % decrease in grain crop yields. This trend is consistent with the experience of major cereal crops such as maize and wheat, which have experienced significant yield reductions on a global scale, totaling 40 megatons annually from 1981 to 2002 due to climate change.

Over the long term, LNTMEAN coefficients negatively influence LNCP, indicating a 1 °C rise in LNTMEAN leads to a 2.349 % decrease in LNCP. This aligns with the findings of [1,37,54,58,60,65,68,71,75,83,120], which consistently demonstrated a negative correlation between LNTMEAN and LNCP. Conversely, this contradicts [63,121], who identified a significant positive correlation between LNTMEAN and LNCP. Additionally, LNTMEAN had no impact on LNCP in Pakistan [50]. In the short term, LNTMEAN positively affects LNCP, predicting a 1 °C increase in LNTMEAN would result in an 8.060 % rise in LNCP.

A slight temperature increase can promote critical stages such as flowering and seed formation, potentially resulting in higher yields. This suggests that, within a certain range, a moderate temperature increase can benefit crop growth. However, beyond this point, the negative consequences of temperature changes tend to outweigh any potential benefits. The maximum temperature coefficients reveal a variety of effects on cereal production. LNTMAX has a short-term unfavorable impact but a long-term good benefit. A 1 °C LNTMAX is expected to reduce LNCP by 0.838 % in the long run and increase LNCP by 4.382 % in the short run. It is corroborated by the findings of the study that [50,53,60,83,122,123] who reported that LNTMAX negatively affects LNCP. The effects of climate change have been most felt by developing nations sectors related to agriculture in recent times. Essential food crops have struggled to adapt to changing weather patterns and planting patterns. Climate change adversities in the agricultural sector include decreased production performance and increased operational costs due to limited resources to combat vulnerabilities. As a result, addressing climate change comes at a high cost, but timely interventions can be implemented to mitigate its negative consequences [75,124].

The ECM_t-1_, shows a highly significant negative impact with a coefficient of −1.795 and a p-value of 0.000, indicating its role in restoring the equilibrium relationship between the variables. The model's goodness of fit is underscored by the high R-squared value of 0.999 and the adjusted R-squared of 0.997, implying a strong explanatory power. The F-statistic of 213.114 further signifies the overall significance of the model. Additionally, the Durbin-Watson statistic of 1.49 indicates the presence of positive autocorrelation.

### 4.7 Diagnostic testing

**Fig. 3:**
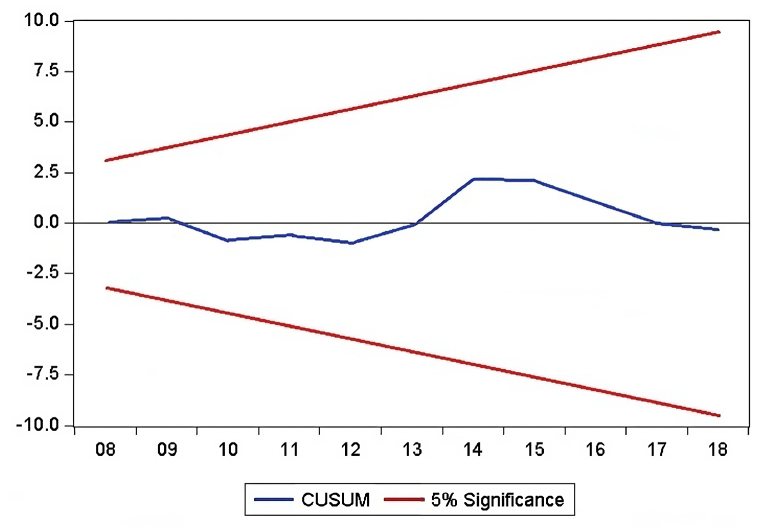
CUSUM test.

**Fig. 4:**
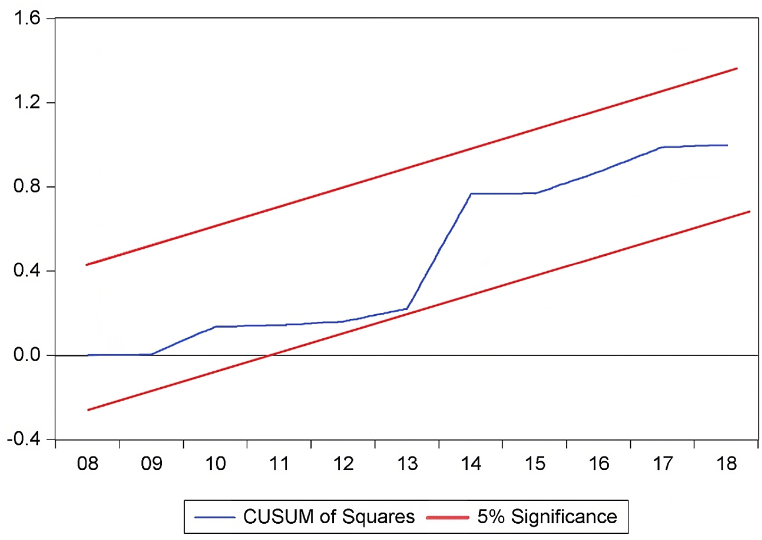
CUSUM square test.

The findings of diagnostic statistical tests conducted to judge the reliable and precise nature of the analysis are shown in Table 9. With a value of 2.41 and a probability of 0.299, the Normality Test of Jarque-Bera indicates that the distribution of the data may be close to being normal. The LM Test for Breusch-Godfrey Serial Correlation yields a statistic of 1.407 with a corresponding probability of 0.294, indicating that there may not be significant serial correlation in the model. Breusch-Pagan-Godfrey, ARCH, Glejser, and Harvey heteroskedasticity tests all produce values of 0.651, 0.264, 0.907, and 1.167, respectively, with associated probabilities that do not show any discernible heteroskedasticity. The correct specification of the model is confirmed by the Ramsey Reset test. The stability of the ARDL model parameters is confirmed by the CUSUM and CUSUMSQ tests, as depicted in Fig. 3, Fig. 4.

**Table 9:** Diagnostic statistics test.

**Particulars**	**F-statistic**	**Prob. F**
Jarque-Bera Normality Test	2.41	0.299
Breusch-Godfrey Serial Correlation LM Test	1.407	0.294
Heteroskedasticity Test: Breusch-Pagan-Godfrey	0.651	0.844
Heteroskedasticity Test: ARCH	0.264	0.610
Heteroskedasticity Test: Glejser	0.907	0.616
Heteroskedasticity Test: Harvey	1.167	0.413
Ramsey Reset Test	0.083	0.778
CUSUM	Stable	
CUSUMSQ	Stable

### 4.8 Robustness estimates

After confirming the variables' long-run cointegration, we examined the variables' long-run coefficients. To make sure the model is robust; we estimated the parameter's long-run coefficients using FMOLS and CCR techniques. The result of the CCR and FMOLS models is presented in Table 10, and it demonstrates that all the variables are significant over the long term. The long-run covariance estimate was calculated in the robustness analysis using parameters to increase the accuracy of the findings. The estimation method involved pre-whitening with a maximum lag of three, as determined by the AIC. During the estimation process, the Bartlett kernel and a fixed Newey-West bandwidth of 4.0 were both used.

**Table 10:** Robustness check.

**Variables**	**Fully Modified Least Squares (FMOLS)**			**Canonical Cointegrating Regression (CCR)**		
	**Coeff.**	**S.E.**	**Prob.**	**Coeff.**	**S.E.**	**Prob.**
LNCA	0.941***	0.025	0.000	0.562***	0.039	0.000
LNTCA	0.734***	0.071	0.000	−0.699***	0.122	0.000
LNFC	−0.026**	0.011	0.017	−0.035**	0.014	0.018
LNPC	0.022***	0.008	0.014	0.050***	0.012	0.000
LNPREC	−0.077***	0.016	0.000	0.138***	0.030	0.000
LNTMIN	33.296***	1.899	0.000	84.019***	3.192	0.000
LNTMEAN	−85.406***	4.950	0.000	−224.546***	8.533	0.000
LNTMAX	51.063***	3.087	0.000	138.239***	5.426	0.000
C	−0.001	0.001	0.604	0.017***	0.002	0.000
**R**^**2**^	**0.904**			**0.884**		
**Adj R**^**2**^	**0.888**			**0.851**		

**Note:** *, **, *** exhibits significance at 10 %, 5 %, 1 % level.

The variables such as LNCA, LNTCA, LNPC, LNTMIN, and TMAX in FMOLS and LNCA, LNPC, LNPREC, LNTMIN, and TMAX in CCR have statistically significant at 1 % level of significance with positive effects on cereal production. LNFC, LNPREC and LNTMIN exhibit a detrimental effect on cereal production reducing it by approximately 0.026 %, 0.077 % and 85.406 % respectively in FMOLS for a 1 % increase in these variables in the long run. On the other hand, LNTCA, LNFC and LNTMIN have a negative impact on cereal production in the CCR model, with a reduction of 0.699, 0.035 and 224.546 % respectively, for a 1 % increase in these variables in the long run. With R-squared values for FMOLS and CCR of 0.904 and 0.884, respectively, both models exhibit high levels of explanatory power and the capacity to fully explain the variation in the dependent variable. Thus, both the CCR and FMOLS models validate that the ARDL model is accurately specified and estimated.

## 5 Conclusion

This study's main goal was to investigate the complex interactions between India's cereal production, cereal area, total cropped area, and various climatic and agricultural factors from 1961 to 2018. This study aimed to answer two important questions: first, whether cereal production in India could be negatively impacted by the looming threat of climate change, and second, whether cereal production trends were significantly influenced by the complex web of agricultural variables, including fertilizer use, pesticide use, and total cropped area. The ARDL model was used to shed light on these questions, allowing for a thorough evaluation of the long-term effects of climate change and agriculture-related variables on cereal production. However, the results of the ARDL bound test for cointegration confirmed that cointegration existed between the variables, which prompted additional research. There are enduring connections between climatic variables, agricultural inputs, and cereal production in both the long run and the short run, according to the computed F-statistic of 6.46, which exceeds the value of the upper bound F > I (1). The findings revealed a complex landscape in this setting. LNCA became a key factor in rising cereal production, as evidenced by its positive coefficient. LNFC and LNPREC had similar positive effects on production trends. LNTCA and LNTMIN, on the other hand, seemed to have little influence over the results of production. The short-run coefficients also shed light on the dynamics that underlie transient fluctuations. LNCA, LNPC, and LNPREC were identified as key factors that temporarily altered cereal production and had statistical significance. The negative coefficient connected to LNFC, on the other hand, suggested a potential short-term decrease in production due to changes in fertilizer consumption. ECM_t-1_ presence confirmed the idea of enduring interconnections by highlighting the constancy of the long-term relationships among the variables. The long-term findings were also supported using econometric tools CCR and FMOLS. These methods confirmed that variations in cereal production in India were significantly influenced by climate as well as agricultural inputs and factors.

## 6 Policy implications

It is essential for policymakers to give preventive measures top priority because the findings highlighted the potential vulnerability of cereal production to climate change. This problem requires a multifaceted approach to be solved. The first viable solution, serving both environmental preservation and cereal production optimization, is the reduction of pesticide and fertilizer usage. The negative environmental effects caused by excessive chemical use can be reduced by adopting integrated pest management strategies and organic farming practices. In addition, due to the importance of rice as a cereal crop, irrigation technology investments are crucial for ensuring a sufficient water supply during times of unpredictable rainfall. Considering shifting climatic patterns, water management strategies can increase crop productivity as well as water use effectiveness.

Additionally, it becomes strategic imperative to promote crop varieties that are climate resilient and can withstand water and temperature stress. To combat the problems brought on by climate change, research and development efforts should concentrate on breeding high-yielding, drought-tolerant, and disease-resistant varieties. Adopting sustainable land management techniques, for instance, conservation agriculture and agroforestry, can improve ecosystem resilience overall and improve soil health and water conservation. To address the complex climate change's effects on cereal production in India, a comprehensive strategy that incorporates sustainable practices, technological innovation, and strong policy frameworks is essential. The results highlight the need for proactive measures to lessen the adverse effects of climate change, ensure sustainable food production, and increase India's agricultural sector's resilience.

## 7 Limitations of the study and future research focus

Limitations of this study include the focus primarily on aggregated national-level data, which may overlook regional disparities and nuances that affect cereal production differently across India. Additionally, the study's reliance on econometric models like ARDL, CCR, and FMOLS assumes linear relationships and may not fully capture non-linear dynamics or interactions among variables. Future research could explore finer geographical resolutions to better understand regional variations in climate impacts on cereal production. Incorporating more detailed data on specific agricultural practices and innovations, such as precision farming techniques or crop diversification strategies, could also provide deeper insights into adaptation and mitigation strategies. Furthermore, longitudinal studies tracking the implementation and effectiveness of policy interventions aimed at climate resilience in agriculture would contribute valuable empirical evidence for informed policymaking.

## Data availability statements

Data will be made available on reasonable request.

## CRediT authorship contribution statement

**Arshdeep Singh:** Writing – review & editing, Writing – original draft, Visualization, Validation, Supervision, Software, Project administration, Methodology, Investigation, Formal analysis, Data curation, Conceptualization. **Kashish Arora:** Writing – review & editing, Data curation. **Suresh Chandra Babu:** Writing – review & editing, Supervision, Project administration, Funding acquisition.

## Declaration of competing interest

The authors declare that they have no competing interests.
